# Trends in Prevalence, Awareness, Treatment and Control of Hypertension during 2001-2010 in an Urban Elderly Population of China

**DOI:** 10.1371/journal.pone.0132814

**Published:** 2015-08-04

**Authors:** Lei Wu, Yao He, Bin Jiang, Dongling Sun, Jianhua Wang, Miao Liu, Shanshan Yang, Yiyan Wang

**Affiliations:** 1 Department of Epidemiology, Institute of Geriatrics, Chinese People's Liberation Army General Hospital, Beijing, China; 2 Beijing Key Laboratory of Aging and Geriatrics, Chinese People's Liberation Army General Hospital, Beijing, China; 3 State Key Laboratory of Kidney Disease, Chinese People's Liberation Army General Hospital, Beijing, China; 4 Department of Acupuncture, Chinese People's Liberation Army General Hospital, Beijing, China; 5 Department of Neuroepidemiology, Beijing Neurosurgical Institute, Capital Medical University, China; Shanghai Institute of Hypertension, CHINA

## Abstract

**Objective:**

As the most important risk factors of cardiovascular disease, pre-hypertension and hypertension are important public health challenges. Few studies have focused on the trends of pre-hypertension and hypertension specifically for the aging population in China. Given the anticipated growth of the elderly population in China, there is an urgent need to document the conditions of pre-hypertension and hypertension in this aging population.

**Methods:**

We conducted two cross-sectional surveys of Chinese adults aged ≥60 years in 2001 and 2010. A total of 2,272 (943 males, 1,329 females) and 2,074 (839 males, 1,235 females) participants were included in the two surveys, respectively.

**Results:**

The age- and sex-standardized prevalence of hypertension significantly increased from 60.1% to 65.2% from the 2001 to the 2010 survey. Among the participants with hypertension, the awareness, treatment and control of hypertension all significantly increased from 69.8% to 74.5%, 50.3% to 63.7%, and 15.3% to 30.3%, respectively, from 2001 to 2010. A logistic regression showed that a higher education level, a higher BMI, a family history of hypertension and doctor-diagnosed cardiovascular disease were significantly associated with hypertension awareness and treatment.

**Conclusion:**

Hypertension prevalence increased rapidly between the years surveyed. Although the awareness, treatment and control of hypertension improved significantly, the values of these variables remained low. More attention should be given to the elderly because the population is aging worldwide, and urgent action, optimal treatment approaches and proper public health strategies must be taken to prevent and manage hypertension.

## Introduction

According to a WHO report, cardiovascular disease (CVD) is estimated to become the leading cause of morbidity and mortality worldwide by 2020 [1]. As the risk factors of CVD, pre-hypertension and hypertension are important public health challenges worldwide [2, 3]. Therefore, an effective strategy for preventing CVD requires increased prevention of hypertension along with timely diagnosis and appropriate treatment of hypertension [4].

A large number of studies have been conducted to evaluate the prevalence, awareness, treatment and control of hypertension among the Chinese [5–12], but data on recent trends of pre-hypertension and hypertension prevalence are rare in China [13–16]. In developed countries such as the US, studies have reported trends of hypertension prevalence, particularly for older US adults [17, 18]. However, few studies have focused on the trends of pre-hypertension and hypertension specifically for the aging population in urban areas of China. The aging population presents a serious challenge for China; individuals aged ≥60 and ≥80 years will constitute 29.7% and 7.6% of the total Chinese population by 2050, respectively [19]. Given the anticipated growth of the elderly population in China, there is an urgent need to document the conditions of pre-hypertension and hypertension in this population.

As the capital of China, Beijing is highly developed and urbanized. To explore the situation of pre-hypertension and hypertension among the aging population during the past decade of rapid development and change, the current study reported the prevalence of pre-hypertension and the prevalence, awareness, treatment and control of hypertension in participants aged ≥60 years in two cross-sectional surveys that were conducted in 2001 and 2010 in Beijing, China.

## Methods

### Study design

As described in our previous study [20], a two-stage stratified sampling method was conducted to recruit participants aged 60 years and older in the Wanshoulu Community within the Haidian District, a metropolitan area that was representative of the geographic and economic characteristics of Beijing in 2001. First, we used the randomized cluster sampling method to randomly select 9 of the 94 communities in the Wanshoulu Community. Second, all of the households with elderly residents were selected from the 9 communities, and one elderly resident from each household was selected. A total of 2,680 elderly individuals were selected and invited to participate in our survey. A total of 2,334 participants completed the survey (the response rate was 87.1%). After excluding 62 people with incomplete data, a total of 2,272 participants (943 males and 1,329 females) were included in our survey in 2001.

In 2010, we conducted a second cross-sectional survey in the same district using the same method as with the 2001 survey. In the second survey, 2,162 of 2,510 elderly participants completed the survey (the response rate was 86.1%). After excluding 88 people with incomplete data, a total of 2,074 participants (839 males and 1,235 females) were included in our 2010 survey. After the two surveys were completed, we found that a total of 731 participants (33%) were included in both surveys.

This study was approved by the Independent Ethics Committee of the Chinese People's Liberation Army General Hospital; signed informed consent was obtained from all the participants.

### Data collection and measurement

Each participant completed a face-to-face interview and a standardized questionnaire on demographic characteristics such as age, gender, marital status, medical history, family history of chronic disease and lifestyle characteristics. Trained observers measured each participant’s height, weight, waist circumference and blood pressure according to the standardized protocol. Height was measured in meters (without shoes), and weight was measured in kilograms (heavy clothing was removed, and one kilogram was deducted for remaining garments). Waist circumference was measured midway between the lower rib margin and iliac crest while participants were in the standing position [21]. Cigarette smoking was defined as having smoked at least one cigarette per day for more than one year [22]. Alcohol consumption was defined as drinking alcohol at least 12 times during the past year [23]. Body mass index (BMI) was calculated as weight in kilograms divided by height in meters squared [21]. The participants were stratified into the following four age groups: 60 to 64, 65 to 69, 70 to 74 and ≥75 years. Overnight fasting blood specimens were obtained for the measurement of serum lipids and glucose, and the samples were sent to the central certified laboratory of the Chinese PLA General Hospital within 30 minutes.

### BP measurement and definitions

The participants were advised to avoid alcohol, cigarette smoking, coffee or tea and exercise prior to blood pressure measurement. Two measurements were obtained from the right arm using standardized mercury sphygmomanometers while participants were in a sitting position [24]. Systolic blood pressure (SBP) and diastolic blood pressure (DBP) were defined as the averages from the two readings. If the two measurements differed by over 10 mmHg, the observers measured the blood pressure a third time and calculated the average of the three measurements as the final measurement.

Based on the measured blood pressure, the participants were classified into the following three groups: optimal blood pressure, pre-hypertension, and hypertension. Optimal blood pressure was defined as a mean SBP <120 mmHg and DBP <80 mmHg. Pre-hypertension was defined as SBP ≥120 mmHg and <140 mmHg or DBP ≥80 mmHg and <90 mmHg. Hypertension was defined as SBP ≥140 mmHg and/or DBP ≥90 mmHg and/or the self-reported use of antihypertensive medication in the previous two weeks [25–27]. Among the participants who were defined as hypertensive in the review, awareness of hypertension was defined as any prior diagnosis of hypertension by a health care professional, treatment of hypertension was defined as use of prescribed antihypertensive medication within the previous two weeks, and control of hypertension was defined as an average SBP <140 mmHg and an average DBP <90 mmHg and was associated with pharmacological treatment of hypertension [28]. The Eighth Joint National Committee (JNC-8) proposed the treatment blood pressure goal of less than 150/90 mmHg among individuals aged 60 years or older [29]. We defined the control of hypertension as SBP <150 mmHg and/or DBP <90 mmHg in a subsequent sensitivity analysis.

### Statistical analysis

The data were entered (double entry) using Epidata (3.1) and analyzed using SPSS (Inc., Chicago, IL, USA) for Windows (19.0). A two-sided P-value of <0.05 was considered statistically significant.

The prevalence of pre-hypertension and the prevalence, awareness, treatment and control of hypertension were standardized by age and sex. We weighted the survey data, and age- and sex-standardizations (both treated as the dichotomous variables) were performed based on the urban population distributions in Beijing in 2001 and 2010 [30]. The baseline characteristics were described using descriptive statistics, and t-tests and chi-square tests were used to examine differences in the continuous and categorical variables, respectively. Prevalence in the two surveys from 2001 and 2010 was compared using a chi-square test. A logistic regression was used to calculate the factors associated with the awareness, treatment and control of hypertension in the 2001 and 2010 surveys.

We conducted sensitivity analyses of subject characteristics and the prevalence, awareness, treatment and control of hypertension during 2001–2010 after excluding those individuals who were included in both surveys (n = 731).

## Results

As shown in Table 1, a total of 2,272 (943 males and 1329 females) and 2,074 (839 males and 1235 females) participants completed the surveys in 2001 and 2010, respectively. The mean age of the participants was 67.92±5.76 years in 2001 and significantly increased to 71.69±6.56 years in 2010. The proportion of participants aged 75 and older had increased rapidly over the decade, which indicated the aging of the population. SBP and DBP also significantly increased from 137.14±21.27 mmHg to 138.93±19.78 mmHg and 77.03±10.52 mmHg to 77.30±9.93 mmHg, respectively, from 2001 to 2010.

**Table 1:** Characteristics of the subjects who completed the surveys in 2001 and 2010.

Characteristics	2001 (n = 2272)	2010 (n = 2074)	P-value
Mean±SD			
Age (year)	67.92±5.76	71.69±6.56	<0.001
Height (m)	1.61±0.08	1.60±0.08	0.388
Weight (kg)	66.70±10.85	64.39±10.80	0.771
BMI (kg/m^2^)	25.62±3.53	25.00±3.41	0.250
Waist (cm)	87.70±9.35	88.32±9.07	0.400
Hip (cm)	101.20±8.01	98.50±7.64	0.330
SBP (mmHg)	137.14±21.27	138.93±19.78	<0.001
DBP (mmHg)	77.03±10.52	77.30±9.93	0.035
TC (mmol/l)	5.33±1.68	5.27±1.02	0.732
TG (mmol/l)	1.55±1.05	1.67±0.93	0.076
HDL-C (mmol/l)	1.37±0.33	1.41±0.37	<0.001
LDL-C (mmol/l)	3.26±0.84	3.25±0.85	0.407
FPG (mmol/l)	6.12±1.93	6.10±1.67	0.021
Number (%)			
Age			
60-	697 (30.7)	360 (17.4)	<0.001
65-	753 (33.1)	392 (18.9)	
70-	525 (23.1)	589 (28.4)	
75-	297 (13.1)	733 (35.3)	
Male	943 (41.5)	839 (40.5)	0.481
Married	1908 (84.0)	1750 (84.4)	0.719
Education ≥7 years	1310 (57.7)	1496 (72.1)	<0.001
Physical exercise ≥1 (h/d)	1676 (73.8)	1780 (85.8)	<0.001
Current drinker	325 (14.3)	418 (20.2)	<0.001
Current smoker	350 (15.4)	230 (11.1)	<0.001
Family history of hypertension	780 (34.3)	806 (38.9)	0.002
Doctor-diagnosed CVD	1085 (47.8)	656 (31.6)	<0.001

### Prevalence of pre-hypertension and hypertension

Table 2 shows the crude age- and sex-standardized prevalence of optimal blood pressure; pre-hypertension; hypertension; and the awareness, treatment and control of hypertension among females and males in the two surveys from 2001 and 2010.

**Table 2:** Prevalence of pre-hypertension and the prevalence, awareness, treatment and control of hypertension among females and males who completed the surveys in 2001 and 2010.

	Total			Male			Female				
	2001 (n = 2272)	2010 (n = 2074)	P-value	2001 (n = 943)	2010 (n = 839)	P-value	2001 (n = 1329)	2010 (n = 1235)	P-value	P-value*	P-value#
Crude prevalence											
Current											
Optimal	289 (12.7)	171 (8.2)	<0.001	118 (12.5)	71 (8.5)	0.006	171 (12.9)	100 (8.1)	<0.001	0.803	0.767
Pre-hypertension	625 (27.5)	494 (23.8)	0.005	263 (27.9)	228 (27.2)	0.736	362 (27.2)	266 (21.5)	0.001	0.732	0.003
Hypertension	1358 (59.8)	1409 (67.9)	<0.001	562 (59.6)	540 (64.4)	0.039	796 (59.9)	869 (70.4)	<0.001	0.887	0.004
Awareness	953 (70.2)	1058 (75.1)	0.004	387 (68.9)	388 (71.9)	0.277	566 (71.1)	670 (77.1)	0.005	0.373	0.027
Treatment	693 (51.0)	945 (67.1)	<0.001	284 (50.5)	347 (64.3)	<0.001	409 (51.4)	598 (68.8)	<0.001	0.758	0.077
Control	209 (15.4)	417 (29.6)	<0.001	95 (16.9)	168 (31.1)	<0.001	114 (14.3)	249 (28.7)	<0.001	0.194	0.326
Age- and sex-adjusted prevalence											
Current											
Optimal	12.7 (11.3–14.1)	9.3 (8.1–10.6)	<0.001	12.6 (10.5–14.7)	9.9 (7.8–11.9)	0.075	12.8 (11.0–14.6)	9.0 (7.4–10.6)	0.002	0.893	0.468
Pre-hypertension	27.2 (25.4–29.0)	25.5 (23.6–27.4)	0.207	28.0 (25.1–30.9)	29.6 (26.5–32.7)	0.448	26.7 (24.3–29.0)	22.7 (20.4–25.1)	0.021	0.495	<0.001
Hypertension	60.1 (58.1–62.1)	65.2 (63.1–67.2)	0.001	59.4 (56.3–62.6)	60.5 (57.2–63.9)	0.628	60.6 (57.9–63.2)	68.3 (65.7–70.9)	<0.001	0.597	<0.001
Awareness	69.8 (67.4–72.3)	74.5 (72.2–76.8)	0.007	68.7 (64.8–72.6)	72.0 (68.1–75.9)	0.231	70.6 (67.5–73.8)	76.0 (73.1–78.9)	0.013	0.464	0.108
Treatment	50.6 (47.9–53.2)	66.4 (63.9–68.9)	<0.001	50.3 (46.2–54.5)	63.7 (59.5–67.9)	<0.001	50.7 (47.3–54.2)	68.0 (64.9–71.2)	<0.001	0.863	0.100
Control	15.3 (13.4–17.2)	30.0 (27.5–32.4)	<0.001	16.9 (13.8–20.0)	31.1 (27.0–35.1)	<0.001	14.2 (11.8–16.6)	29.3 (26.2–32.4)	<0.001	0.187	0.486

* 2001: male vs. female

^#^ 2010: male vs. female

From 2001 to 2010, the proportion of the population with optimal blood pressure decreased significantly, and the age- and sex-standardized prevalence of pre-hypertension decreased from 27.2% to 25.5%, particularly in females (26.7% to 22.7%, P = 0.021). Additionally, the prevalence of hypertension significantly increased from 60.1% to 65.2% (P = 0.001), particularly in females (60.6% to 68.3%, P<0.001). Males had a higher prevalence of pre-hypertension (29.6% for males vs. 22.7% for females in 2010, P<0.001), and females had a higher prevalence of hypertension (60.5% for males vs. 68.3% for females in 2010, P<0.001), particularly in 2010. There were no significant sex differences in pre-hypertension and hypertension prevalence in 2001, but the trend was similar to that in 2010.

**Fig 1:**
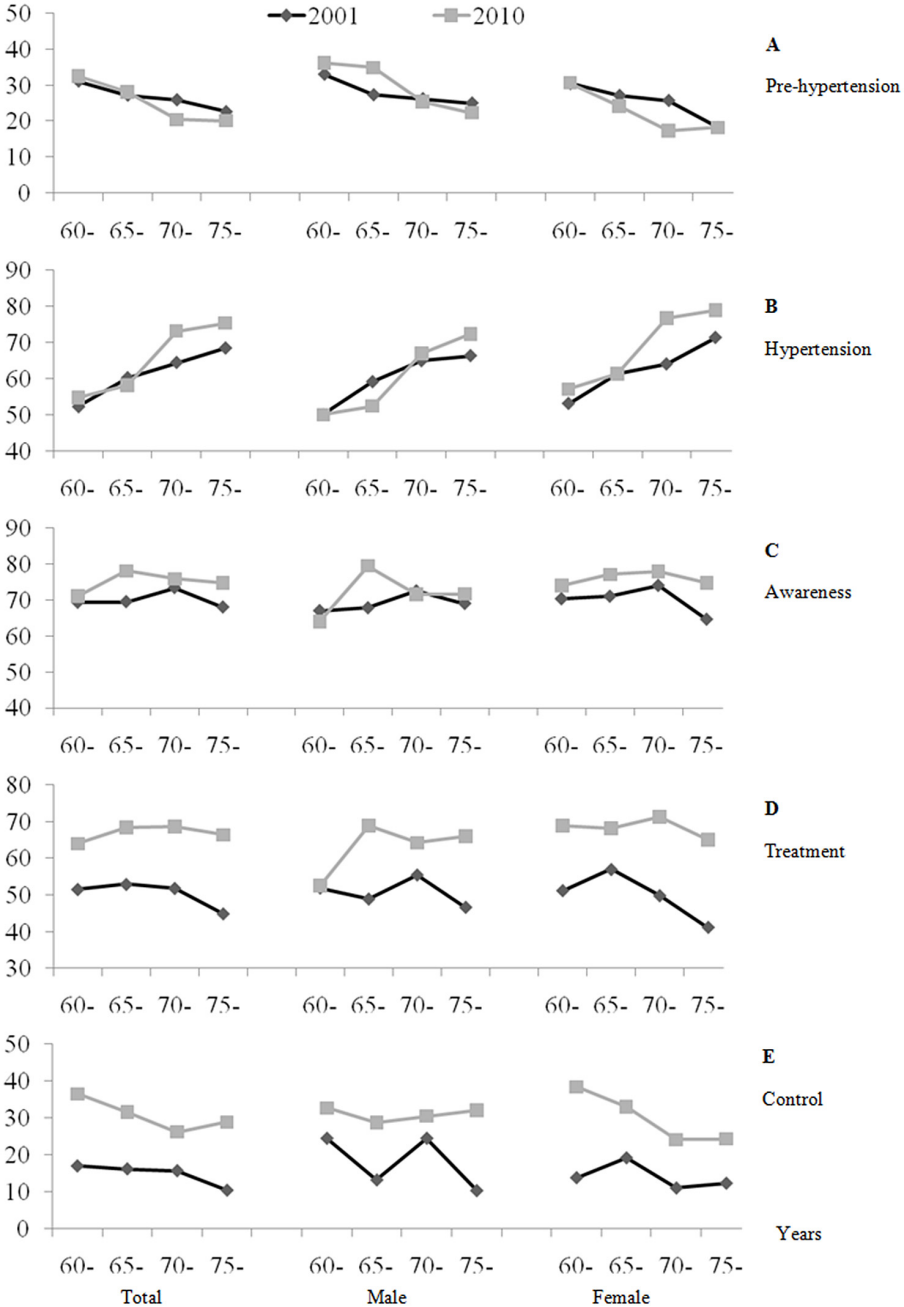
Age-specific prevalence of pre-hypertension and the prevalence, awareness, treatment and control of hypertension. Fig 1 shows the age-specific prevalence of (A). prehypertension (B). prevalence (C). awareness (D). treatment and (E). control of hypertension.

After stratifying the participants into four age groups, the results indicated that the prevalence of pre-hypertension decreased with age and that hypertension markedly increased with age. The greatest decline and increment were in the subgroup of those aged 65–69 years in both sexes (Fig 1A and 1B). As shown in Fig 1B, the age-specific prevalence of hypertension increased in all age groups from the 2001 to the 2010 survey, except for those aged 65–69 years. From 2001 to 2010, the prevalence of hypertension was highest among the subgroup of participants aged ≥70 years in both sexes.

### Awareness, treatment and control of hypertension

Among the participants who had hypertension, 69.8% were aware of their condition in 2001, and this rate significantly increased to 74.5% in 2010 (P = 0.007), particularly in females (from 70.6% to 76.0%, P = 0.013).

From 2001 to 2010, there was a significant increase in hypertensive patients receiving treatment both in males (50.3% to 63.7%, P<0.001) and females (50.7% to 68.0%, P<0.001).

In 2001, only 15.3% of patients with hypertension had controlled their blood pressure to reach the optimal range, but in 2010, this proportion approximately doubled to 30.3% (31.1% for males and 29.3% for females, both P<0.001).

The age-specific subgroups in both sexes indicated that the participants aged 60–64 years and ≥75 years had both the lowest hypertension awareness and treatment rates in both surveys (Fig 1C and 1D). In 2010, the participants aged 60–64 years had the highest hypertension control rate (Fig 1E).

### Factors associated with awareness, treatment and control of hypertension

The logistic regression model analysis results of factors associated with hypertension awareness, treatment and control in 2001 and 2010 are shown in Table 3. A higher education level, a higher BMI, a family history of hypertension and doctor-diagnosed CVD were all significantly associated with hypertension awareness and treatment in both 2001 and 2010. A lower age, a higher education level and doctor-diagnosed CVD were significantly associated with hypertension control in 2001. We also found that a lower BMI and a family history of hypertension were significantly associated with hypertension control in 2010.

**Table 3:** Factors associated with the awareness, treatment and control of hypertension in two surveys, 2001 and 2010.

	Awareness		Treatment		Control	
	2001 (n = 1358) OR (95% CI)	2010 (n = 1409) OR (95% CI)	2001 (n = 1358) OR (95% CI)	2010 (n = 1409) OR (95% CI)	2001 (n = 1358) OR (95% CI)	2010 (n = 1409) OR (95% CI)
Age						
60-	1.00	1.00	1.00	1.00	1.00	1.00
65-	1.04 (0.75–1.45)	1.57 (0.99–2.49)	1.15 (0.85–1.55)	1.36 (0.89–2.08)	0.88 (0.60–1.30)	0.83 (0.55–1.25)
70-	1.28 (0.90–1.84)	1.38 (0.91–2.08)	1.09 (0.79–1.51)	1.45 (0.98–2.13)	0.89 (0.58–1.35)	0.64 (0.44–0.94)*
75-	1.10 (0.72–1.69)	1.29 (0.85–1.94)	0.90 (0.61–1.34)	1.30 (0.89–1.91)	0.54 (0.31–0.96)*	0.72 (0.49–1.04)
P for trend	0.53	0.27	0.46	0.30	0.21	0.13
Female	1.06 (0.79–1.42)	1.22 (0.90–1.65)	1.10 (0.84–1.42)	1.25 (0.95–1.66)	0.87 (0.61–1.23)	0.92 (0.70–1.22)
Married	0.92 (0.65–1.30)	0.80 (0.55–1.16)	0.90 (0.66–1.24)	0.87 (0.63–1.22)	1.24 (0.78–1.97)	0.80 (0.57–1.12)
Education ≥7 years	1.36 (1.04–1.79)*	1.32 (0.98–1.77)	1.93 (1.51–2.47)***	1.73 (1.32–2.26)***	1.43 (1.02–1.99)*	1.75 (1.31–2.33)***
Physical exercise ≥1 (h/d)	1.11 (0.84–1.46)	0.82 (0.56–1.20)	1.04 (0.81–1.34)	0.82 (0.58–1.16)	1.02 (0.73–1.44)	0.93 (0.66–1.30)
BMI (kg/m^2^)	1.10 (1.06–1.14)***	1.05 (1.01–1.09)*	1.08 (1.05–1.12)***	1.02 (0.99–1.06)	1.01 (0.97–1.06)	0.95 (0.92–0.99)**
Current smokers	0.82 (0.57–1.17)	1.07 (0.70–1.64)	0.81 (0.58–1.14)	0.82 (0.56–1.21)	1.30 (0.85–1.99)	0.74 (0.48–1.14)
Current drinker	0.91 (0.63–1.31)	0.79 (0.56–1.11)	0.86 (0.61–1.20)	0.99 (0.72–1.36)	0.73 (0.46–1.16)	1.17 (0.85–1.62)
Family history of hypertension	2.07 (1.59–2.70)***	2.37 (1.80–3.12)***	1.58 (1.25–1.99)***	2.22 (1.74–2.84)***	1.05 (0.77–1.42)	1.36 (1.07–1.72)*
Doctor diagnosed CVD	2.43 (1.90–3.12)***	2.55 (1.90–3.43)***	1.79 (1.43–2.25)***	2.26 (1.75–2.93)***	1.75 (1.28–2.40)***	1.52 (1.19–1.94)**

*** P<0.001;

** P<0.01;

* P<0.05

### Sensitivity analysis

After excluding the data of the 731 participants who were included in both surveys, we assessed the subject characteristics and the prevalence, awareness, treatment and control of hypertension in 2001 and 2010 (S1 and S2 Tables), and the results were similar to those reported above. According to the new treatment blood pressure goal (JNC-8) of the elderly participants [29], the control rate of hypertension significantly increased from 25.3% in 2001 to 36.9% in 2010 (S3 Table).

## Discussion

To our knowledge, the present study is the first to report the trends of pre-hypertension prevalence and hypertension prevalence, awareness, treatment and control in the past decade in an elderly population in Beijing, China. Two cross-sectional surveys were conducted with participants aged ≥60 years in the same district and using the same methods in both 2001 and 2010. The results showed that the age- and sex-standardized prevalence of pre-hypertension decreased and that the prevalence, awareness, treatment and control of hypertension all increased significantly from 2001 to 2010.

The upward trends in age- and sex-standardized prevalence of hypertension (from 60.1% in 2001 to 65.2% in 2010) found in the current study are consistent with the results of previous studies in China. Among participants aged ≥60 years, Xi et al. reported that the prevalence of hypertension increased from 48.4% in 1991 to 53% in 2009 in nine provinces in China [16]. Zhao et al. reported that the prevalence of hypertension increased from 42.2% in 1999 to 70.8% in 2007 in Shandong Province [13], and the prevalence of hypertension in a northern Chinese population increased from 68.6% in 1991 to 71.2% in 2011 [14]. However, the prevalence of hypertension in US elderly individuals has been relatively stable over the past decade [31, 32], and the prevalences of hypertension in the present study of participants aged ≥75 years (75.4%) and ≥60 years (65.2%) in 2010 are similar to those reported in the US. Bromfield et al. reported that 76.5% of US adults ≥80 years had hypertension in 2005–2010 [17], and Guo et al. reported that the prevalence of hypertension was 66.7% among US adults ≥60 years in 2009 and 2010 [31].

From 2001 to 2010, the prevalence of pre-hypertension decreased, whereas the prevalence of hypertension increased with increasing age, which is consistent with other studies [13, 15 33, 34]. Males had a higher prevalence of pre-hypertension but a lower prevalence of hypertension, particularly in 2010, demonstrating a trend similar to that reported for the subgroup aged 65–74 by Gu et al. [28]. These results indicate that females in the aging population might have an increased chance of developing hypertension if they are pre-hypertensive. Whether gender influences the progression of pre-hypertension to hypertension among elderly people might be an interesting topic to explore with a larger sample size in the future.

Among the participants with hypertension, the awareness, treatment and control of hypertension all significantly increased from 69.8% to 74.5%, 50.3% to 63.7%, and 15.3% to 30.3%, respectively, from 2001 to 2010. These rates are higher than those of Xi et al [16], who reported increases of 38.7% to 54.3% for awareness, 30.5% to 49.0% for treatment, and 6.1% to 12.0% to control from 2000 to 2009 among participants aged ≥60 years. The rates found in the present study are also higher than those of Zhao et al [15], who reported increases of 39.1% to 49.2% for awareness, 28.1% to 43.4% for treatment, and 4.4% to 7.1% for control from 1999 to 2007 among all age groups. Furthermore, the rates of the present study are higher than those of a 2007 southeast Asian study of 19,848 participants aged ≥65 years [35]. Both the increase in the education level of our population in the past decade (from 57.7% to 72.1%) and preventive measures from the government and health professionals during this time might have contributed to the increases in awareness, treatment and control of hypertension. However, the prevalence remained lower than in the US elderly population (73.4% to 84.0% for awareness, 72.4% to 85.3% for treatment, and 34.1% to 54.9% for control from 2001 to 2010) [31] and in participants aged ≥65 years in Macau, China (79%, 75% and 35% for awareness, treatment and control, respectively, in 2012) [11]. Given the rapid social and economic changes occurring in China, public health strategies should be developed to meet the need of the aging population in China, particularly by providing more effective pharmacologic interventions aimed at elderly hypertensive patients.

Previous studies have found that older age and a higher BMI are associated with greater hypertension awareness and treatment but poorer hypertension control, which is in accordance with the current report [8, 9, 36–39]. A family history of hypertension is also significantly associated with greater hypertension awareness, treatment and control [8, 9, 40]. Participants who know their high risk of hypertension pay special attention to their blood pressure. Other studies have reported that being female, being retired, being married and cigarette smoking are all related to hypertension awareness, treatment and control, [8, 10, 39], although these variables did not reach statistical significance in the present study. We found that more females than males were both aware of their hypertension condition and received treatment, but the percentage of controlled hypertension was slightly lower among females than among males. The associations between education level and hypertension awareness, treatment and control are inconsistent among previous studies. A multi-ethnic Asian population study [39] and Wang et al. [9] reported that high education levels were associated with poor hypertension awareness and treatment, but Tian et al. [33] reported that increased awareness and treatment were found among those with high education levels, which is consistent with the findings of the present study. The differences among populations might be partially attributed to differences in education level; thus, a larger sample size is required to detect more factors associated with hypertension in future research. We also found that diagnosed CVD was significantly associated with hypertension awareness, treatment and control, indicating that the control of hypertension is a key treatment for patients who are diagnosed with CVD and that lowering blood pressure can significantly reduce the risk of cardiovascular events [41].

The age of the urban elderly population increased significantly over the decade evaluated in the current study. We previously found the same result in other areas of China [13–15], which suggests the aging of the population. The percentage of individuals more than 60 years old has dramatically increased during this time, and China has entered into an aging society [42, 43]. With the increased aging of the population, China is facing many challenges in adequately meeting the medical demand for chronic disease treatment [44]. The prevalence of multiple chronic conditions (MCC) among individuals increases with age: the older the population, the greater the prevalence of MCC [45]. In the present study, we found that elderly people aged 60–64 years had fewer coexisting diseases than did those greater than 65 years old (data not shown), the latter of which increases the difficulty of treatment and control of hypertension. This may explain the higher control rate of hypertension in the relatively younger elderly population.

The present study has some limitations. First, because of the characteristics of cross-sectional studies, we could only observe the prevalence of hypertension at the two survey times and, thus, cannot infer causation. Second, we did not collect detailed information on diet, such as the amount of salt consumed. Therefore, we were unable to detect relationships between diet conditions and hypertension prevalence rates. Third, we measured blood pressure during a single visit, which might overestimate or underestimate the true prevalence of hypertension [15]. Fourth, we found that the mean age of participants in 2010 was significantly higher than that in 2001 and that the prevalence of hypertension significantly increased with age. Although the age-specific prevalence of hypertension approximately increased in all age groups, age might also be a confounding factor in the present study. Although we adjusted several potential confounding variables in the current analysis, differences between surveys in age and other unmeasured covariates might have affected the results of the present study. As Beijing is the capital of China, residents of this city might be more educated and wealthy than those in rural areas, and they might pay more attention to their health. These characteristics could explain the higher hypertension awareness, treatment and control rates in Beijing compared with other areas. Prospective studies with larger sample sizes are required to examine the causes of the trends of pre-hypertension and hypertension prevalence, awareness, treatment and control in the elderly population in the future.

In conclusion, according to two cross-sectional surveys, hypertension prevalence increased rapidly over the past decade among urban elderly residents in Beijing, northern China. Although the awareness, treatment and control of hypertension improved significantly, they remain low. We should pay greater attention to the elderly, as population aging is occurring worldwide. Thus, urgent action, optimal treatment approaches and proper public health strategies are needed for the prevention and management of hypertension, with the ultimate goal of lowering the incidence of hypertension-related chronic diseases.

## Supporting Information

S1 Dataset The dataset of the participants in two surveys. It contains the raw data of 4,346 participants (2,272 participants in 2001 and 2,074 participants in 2010) with 22 variables. Including investigation year (2001, 2010), code, gender (male, female), age, education years (0–6, 7–9, 10–12, 13–16, ≥17 years), marriage status (married, single, divorce, widowed), height (m), weight (kg), waist (cm), hip (cm), SBP (mmHg), DBP (mmHg), TC (mmol/l), TG (mmol/l), HDL-C (mmol/l), LDL-C (mmol/l), FPG (mmol/l), physical exercise (< 1 h/d, ≥1 h/d), smoking status, drinking status, family history of hypertension and doctor-diagnosed CVD.
(XLS)

S1 Table Characteristics of the subjects who completed the surveys in 2001 and 2010 (excluding the data of 731 participants that completed both surveys). S1 Table shows the subject characteristics in 2001 and 2010, after excluding the data of the 731 participants who were included in both surveys.
(DOC)

S2 Table Prevalence of pre-hypertension and the prevalence, awareness, treatment and control of hypertension among females and males who completed the surveys in 2001 and 2010 (excluding the data of 731 participants that completed both surveys) S2 Table shows the prevalence, awareness, treatment and control of hypertension in 2001 and 2010, after excluding the data of the 731 participants who were included in both surveys. (DOC)

S3 Table Hypertension control rate of the all participants who completed the surveys in 2001 and 2010 (excluding the data of 731 participants who completed both surveys) (JNC-8). S3 Table shows the control rate of hypertension, according to the new treatment blood pressure goal (JNC-8) of the elderly participants.
(DOC)
