# Supplementary material for: Trends in Prevalence, Awareness, Treatment and Control of Hypertension during 2001-2010 in an Urban Elderly Population of China
Source: PLoS One. 2015 Aug 4;10(8):e0132814. doi: 10.1371/journal.pone.0132814 (PMC4524712; doi:10.1371/journal.pone.0132814)
Supplement: S1 Table — shows the subject characteristics in 2001 and 2010, after excluding the data of the 731 participants who were included in both surveys. (DOC) [file pone.0132814.s002.doc]

**S1 Table. Characteristics of the subjects who completed the surveys in 2001 and 2010 (excluding the data of 731 participants that completed both surveys).**

| **Characteristics** | **2001 (n=1541)** | **2010 (n=1343)** | **P-value** |
| --- | --- | --- | --- |
| Mean±SD |  |  |  |
| Age (year) | 68.03±5.85 | 70.15±6.57 | <0.001 |
| Height (m) | 1.61±0.08 | 1.60±0.08 | 0.460 |
| Weight (kg) | 66.86±10.93 | 64.06±10.70 | 0.481 |
| BMI (kg/m^2^) | 25.73±3.59 | 24.91±3.36 | 0.102 |
| Waist (cm) | 88.12±9.34 | 87.94±8.94 | 0.242 |
| Hip (cm) | 101.36±7.98 | 98.07±7.43 | 0.126 |
| SBP (mmHg) | 138.27±21.88 | 139.62±19.58 | <0.001 |
| DBP (mmHg) | 76.91±10.43 | 77.15±10.06 | 0.069 |
| TC (mmol/l) | 5.31±1.00 | 5.22±1.02 | 0.151 |
| TG (mmol/l) | 1.55±1.05 | 1.66±0.95 | 0.148 |
| HDL-C (mmol/l) | 1.37±0.33 | 1.41±0.37 | <0.001 |
| LDL-C (mmol/l) | 3.26±0.84 | 3.21±0.85 | 0.352 |
| FPG (mmol/l) | 6.12±1.96 | 6.08±1.63 | 0.011 |
| Number (%) |  |  |  |
| Age |  |  |  |
| 60- | 464 (30.1) | 214 (15.9) | <0.001 |
| 65- | 515 (33.4) | 224 (16.7) |  |
| 70- | 350 (22.7) | 405 (30.2) |  |
| 75- | 212 (13.8) | 500 (37.2) |  |
| Male | 629 (40.8) | 542 (40.4) | 0.802 |
| Married | 1286 (83.5) | 1115 (83.0) | 0.758 |
| Education ≥7 years | 841 (54.6) | 952 (70.9) | <0.001 |
| Physical exercise ≥1 (h/d) | 1139 (73.9) | 1144 (85.2) | <0.001 |
| Current drinker | 242 (15.7) | 144 (10.7) | <0.001 |
| Current smoker | 217 (14.1) | 263 (19.6) | <0.001 |
| Family history of hypertension | 528 (34.3) | 536 (39.9) | 0.002 |
| Doctor-diagnosed CVD | 756 (49.1) | 446 (33.2) | <0.001 |
