# Supplementary material for: Trends in Prevalence, Awareness, Treatment and Control of Hypertension during 2001-2010 in an Urban Elderly Population of China
Source: PLoS One. 2015 Aug 4;10(8):e0132814. doi: 10.1371/journal.pone.0132814 (PMC4524712; doi:10.1371/journal.pone.0132814)
Supplement: S2 Table — (DOC) [file pone.0132814.s003.doc]

|  | **Total** |  |  | **Male** |  |  | **Female** |  |  |  |  |
| --- | --- | --- | --- | --- | --- | --- | --- | --- | --- | --- | --- |
|  | **2001 (n=1541)** | **2010 (n=1343)** | **P-value** | **2001 (n=629)** | **2010 (n=542)** | **P-value** | **2001 (n=912)** | **2010 (n=801)** | **P-value** | **P-value*** | **P-value#** |
| Crude prevalence |  |  |  |  |  |  |  |  |  |  |  |
| Current |  |  |  |  |  |  |  |  |  |  |  |
| Normal | 189 (12.3) | 97 (7.2) | <0.001 | 78 (12.4) | 44 (8.1) | 0.017 | 111 (12.2) | 53 (6.6) | <0.001 | 0.893 | 0.297 |
| Pre-hypertension | 413 (26.8) | 307 (22.9) | 0.015 | 176 (28.0) | 148 (27.3) | 0.797 | 237 (26.0) | 159 (19.9) | 0.003 | 0.385 | 0.001 |
| Hypertension | 939 (60.9) | 939 (69.9) | <0.001 | 375 (59.6) | 350 (64.6) | 0.082 | 564 (61.8) | 589 (73.5) | <0.001 | 0.379 | <0.001 |
| Awareness | 660 (70.3) | 710 (75.6) | 0.009 | 262 (69.9) | 255 (72.9) | 0.374 | 398 (70.6) | 455 (77.2) | 0.010 | 0.818 | 0.130 |
| Treatment | 478 (50.9) | 633 (67.4) | <0.001 | 196 (52.3) | 221 (63.1) | 0.003 | 282 (50.0) | 412 (69.9) | <0.001 | 0.496 | 0.031 |
| Control | 141 (15.0) | 290 (30.9) | <0.001 | 59 (15.7) | 113 (32.3) | <0.001 | 82 (14.5) | 177 (30.1) | <0.001 | 0.616 | 0.474 |
| Age- and Sex-adjusted prevalence |  |  |  |  |  |  |  |  |  |  |  |
| Current |  |  |  |  |  |  |  |  |  |  |  |
| Normal | 12.2 (10.6-13.8) | 8.3 (6.8-9.8) | 0.001 | 12.4 (9.8-15.0) | 9.3 (6.8-11.8) | 0.097 | 12.1 (10.0-14.2) | 7.6 (5.8-9.5) | 0.002 | 0.864 | 0.282 |
| Pre-hypertension | 26.5 (24.3-28.7) | 24.3 (22.0-26.6) | 0.194 | 28.1 (24.5-31.6) | 28.9 (25.0-32.8) | 0.763 | 25.4 (22.5-28.2) | 21.2 (18.3-24.1) | 0.040 | 0.235 | 0.001 |
| Hypertension | 61.3 (58.9-63.8) | 67.4 (64.8-69.9) | 0.001 | 59.6 (55.7-63.4) | 61.7 (57.6-65.9) | 0.433 | 62.6 (59.4-65.7) | 71.2 (68.0-74.3) | <0.001 | 0.223 | <0.001 |
| Awareness | 70.0 (67.1-72.9) | 75.1 (72.2-77.9) | 0.014 | 69.8 (65.2-74.5) | 73.0 (68.1-77.9) | 0.373 | 70.1 (66.3-73.9) | 76.3 (72.8-79.8) | 0.017 | 0.956 | 0.257 |
| Treatment | 50.3 (47.1-53.5) | 66.9 (63.8-70.0) | <0.001 | 52.0 (46.9-57.1) | 62.9 (57.6-68.1) | 0.004 | 49.2 (45.1-53.3) | 69.3 (65.5-73.1) | <0.001 | 0.387 | 0.045 |
| Control | 15.0 (12.7-17.3) | 31.6 (28.5-34.7) | <0.001 | 15.8 (12.1-19.5) | 32.7 (27.5-37.8) | <0.001 | 14.5 (11.6-17.4) | 31.0 (27.1-34.8) | <0.001 | 0.591 | 0.607 |
| * 2001 male vs. female; # 2010 male vs. female | | | | | | | | | | | |

**S2 Table. Prevalence of pre-hypertension and the prevalence, awareness, treatment and control of hypertension among females and males** **who completed the surveys in 2001 and 2010 (excluding the data of 731 participants that completed both surveys)**
