# Supplementary material for: Trends in Prevalence, Awareness, Treatment and Control of Hypertension during 2001-2010 in an Urban Elderly Population of China
Source: PLoS One. 2015 Aug 4;10(8):e0132814. doi: 10.1371/journal.pone.0132814 (PMC4524712; doi:10.1371/journal.pone.0132814)
Supplement: S3 Table — shows the control rate of hypertension, according to the new treatment blood pressure goal (JNC-8) of the elderly participants. (DOC) [file pone.0132814.s004.doc]

**S3 Table. Hypertension control rate of the all participants who completed the surveys in 2001 and 2010 (excluding the data of 731 participants who completed both surveys) (JNC-8).**

|  | **Total** |  |  | **Male** |  |  | **Female** |  |  |  |  |
| --- | --- | --- | --- | --- | --- | --- | --- | --- | --- | --- | --- |
|  | **2001** | **2010** | **P-value** | **2001** | **2010** | **P-value** | **2001** | **2010** | **P-value** | **P-value*** | **P-value#** |
| All participants | (n=2272) | (n=2074) |  | (n=943) | (n=839) |  | (n=1329) | (n=1235) |  |  |  |
| Control of hypertension | 576 (25.4) | 800 (38.6) | <0.001 | 254 (26.9) | 330 (39.3) | <0.001 | 332 (24.2) | 470 (38.1) | <0.001 | 0.144 | 0.558 |
| Age- and Sex-adjusted control of hypertension | 25.3 (23.5-27.1) | 36.9 (34.8-39.0) | <0.001 | 26.8 (24.0-29.7) | 36.6 (33.4-39.9) | 0.016 | 24.3 (22.0-26.6) | 37.1 (34.4-39.8) | 0.001 | 0.174 | 0.816 |
| Excluded reduplicated data | (n=1541) | (n=1343) |  | (n=629) | (n=542) |  | (n=912) | (n=801) |  |  |  |
| Control of hypertension | 375 (24.3) | 545 (40.6) | <0.001 | 161 (25.6) | 220 (40.6) | <0.001 | 214 (23.5) | 325 (40.6) | <0.001 | 0.338 | 0.995 |
| Age- and Sex-adjusted control of hypertension | 24.3 (22.2-26.5) | 39.2 (36.5-41.8) | <0.001 | 25.6 (22.2-29.0) | 38.6 (34.5-42.8) | <0.001 | 23.5 (20.7-26.2) | 39.5 (36.1-42.9) | <0.001 | 0.333 | 0.744 |

* 2001 male vs. female; # 2010 male vs. female
